# Problématique de l'utilisation des Moustiquaires Imprégnées d'insecticide à Longue Durée (MILD) chez les enfants de moins de 5 ans en République Démocratique du Congo

**DOI:** 10.11604/pamj.2016.23.101.7050

**Published:** 2016-03-16

**Authors:** Cilundika Mulenga Philippe, Nyota Nsenga Odile, Oscar Luboya Numbi

**Affiliations:** 1 Faculté de Médecine, Département de Santé Publique, Université de Lubumbashi, Lubumbashi, République Démocratique du Congo

**Keywords:** Paludisme, moustiquaire imprégnée, insecticide, utilisation, Katanga, Malaria, insecticide, impregnated mosquito nets, use, Katanga

## Abstract

**Introduction:**

Le paludisme est une maladie parasitaire à transmission vectorielle qui constitue un problème majeur de santé publique dans les pays tropicaux, particulièrement les pays de l'Afrique Subsaharienne. La présente étude avait pour objectif d'identifier le niveau d'utilisation de la MILD chez les enfants de moins de 5 ans dans la zone de santé de Mumbunda.

**Méthodes:**

L’étude, de type transversal, a été mené du 25 au 27 octobre 2013 et a porté sur un échantillon de 410 ménages tirés au hasard et ayant au moins un enfant de moins de 5 ans.

**Résultats:**

Parmi les ménages qui avaient déclaré possédaient la MILD 13,1% (n=54) des répondant avaient déclaré les utilisé chez les enfants de moins de 5ans et 80,2% pour tout le monde. Dans 22,0% des cas, les répondants ont évoqué le manque d'argent comme motif de la non possession de la MILD. Les répondants savaient déclarer à 79,8% avoir utilisé la MILD pour se protéger contre la malaria et à 66,3% pour se protéger contre les piqures des moustiques. La MILD a été étalée à l'ombre pendant 24h avant d’être utilisée pour la première fois par 77,9% des ménages. Les répondants avaient déclaré à 15,3% (n=63) avoir reçu les conseils par les médias pour l'usage de la moustiquaire. Et le personnel médical était la source la plus importante pour expliquer le mode d'emploi de la MILD pour 51,2% des ménages.

**Conclusion:**

La réussite pour faire reculer le paludisme doit nécessairement passer par la prévention, le suivi et l’évaluation de l'utilisation des moustiquaires imprégnées au niveau de la zone de santé de Mumbunda.

## Abstract (en)

**Introduction:**

Malaria is a vector-borne infectious disease caused by parasites, which represents a major public health problem in tropical countries, especially Sub-Saharan Africa. This study aimed to identify the level of LLIN use in children less than 5 years in Mumbunda health zone.

**Methods:**

The crosscutting study was conducted from 25 to 27 October 2013 and involved a random sample of 410 households with at least a child under 5 years.

**Results:**

Among households reported owning LLIN, 13.1% (n=54) of respondents reported having used it among children under 5 years and 80.2% to everybody. In 22.0% of cases, respondents cited lack of money as the reason for non-possession of LLIN. This study found that 79.8% of respondents declared having used LLIN to protect themselves against malaria and 66.3% to protect themselves against mosquito biking. LLIN was spread under shade for 24h before being used, for the first time, by 77.9% of the households. The respondents reported (15.3%, n=63) receiving information by the media about the use of mosquito net. Healthcare personnel was the most important source to demonstrate the correct way to use LLIN for 51.2% of the households.

**Conclusion:**

The success of Roll Back Malaria must necessarily pass through prevention, follow-up and evaluation of the use of impregnated mosquito nets in Mumbunda health zone.

## Introduction

Le paludisme est une pathologie qui peut être mortelle il est dû à des parasites transmis à l'homme par des piqûres de moustiques infectés [1]. C'est une des maladies communes affectant des êtres humains dans le monde. Son impact majeur est presque entièrement sur les pays en développement, surtout en Afrique. Presque la moitié de la population dans le monde est exposé au risque de contracter la maladie [2]. Le paludisme reste la principale cause de morbidité et selon l'OMS 2 16 millions de cas et 660 000 décès en 2010 et pour la plupart parmi les enfants africains. Plus de 85% des cas et 90% de décès dus au paludisme surviennent en Afrique Subsaharienne.et surtout pendant la saison de pluie [1]. L'Afrique a plusieurs facteurs liés au risque de la maladie. Ces facteurs sont liés à l'existence du vecteur qui favorise la transmission du parasite et à cela il faut ajouter le climat chaud et conditions socio-économiques faibles qui ont un impact sur le contrôle de la maladie. D'autres zones qui sont en danger comprennent quelques pays en Amérique du Sud et l'Asie du sud. Toutefois dans une moindre mesure certaines parties de l'Europe sont également affectés [3]. En 2011, 99 pays étaient confrontés à une transmission continue du paludisme [4]. Les groupes de population les plus spécialement à risque sont [5]: les enfants dans des zones de transmission stable qui n'ont pas encore développé leur immunité; les femmes enceintes non immunisées, chez qui le paludisme entraîne souvent des fausses couches, des décès maternels et de faibles poids de naissance; les femmes enceintes semi-immunisées dans les régions de forte transmission; les femmes enceintes semi-immunisées infectées par le VIH dans les zones de transmission stable ont un risque accru de contracter le paludisme pendant toute leur grossesse; les personnes vivant avec le VIH/sida; les voyageurs internationaux en provenance de régions exemptes de paludisme [6]. Les jeunes enfants dans l'Afrique Subsahariennes est le groupe vulnérable le plus affecté [3]. Le paludisme est également un facteur important d'anémie chez les enfants, affectant gravement la croissance et le développement [7]. Les femmes Enceintes sont en danger parce que leur immunité a été diminué par la grossesse [8]. Les taux de mortalité les plus élevés sont dans les pays avec des proportions plus élevées de leur population vivant dans la pauvreté avec moins de 1,25 dollars par an. Le renforcement des mesures de lutte et de prévention permet de réduire de façon spectaculaire la charge palustre dans certains endroits. Parmi ces mesures figure la moustiquaire imprégnée d'insecticide [9]. Les six pays les plus touchés sont le Nigeria, la République démocratique du Congo(RDC), la Tanzanie, l'Ouganda, le Mozambique, et la Cote d'Ivoire avec 103 millions de personnes. Le continent Asiatique vient en seconde position et le nombre des cas par an, en Inde par exemple est estimé à 24 millions [10]. La RDC et le Nigéria représentent à eux seuls plus de 40% des cas [11]. En RDC, le paludisme figure parmi les principales causes de morbidité et de mortalité. Il est responsable de 10% de décès des nourrissons âgés de moins d'un an [12]. Dans la province du Katanga, 30 à 50% des cas des fièvres sont attribuées au paludisme chez les enfants de moins de 5 ans [13].

Le paludisme constitue ainsi donc un problème majeur de santé publique en RDC. Les taux élevés de morbidité et de mortalité peuvent être expliqués par plusieurs facteurs suivants: l'insalubrité du milieu, les constructions anarchiques, l'ignorance par la population des méthodes de prévention, leur faible participation aux activités de lutte, l'absence de la protection individuelle contre les moustiques, la résistance croissante du plasmodium aux antipaludiques usuels [14]. Cependant la problématique liée à la couverture après les interventions de distribution gratuite des moustiquaires imprégnées d'insecticide ainsi que son utilisation chez les enfants de moins de 5 ans persiste [15] et beaucoup d'auteurs l'ont abordée d'une manière ou d'une autre. L'utilisation de la MILD est associée à une réduction de 44% de mortalité. Ce niveau de protection correspond à environ sept décès évités pour 1000 moustiquaires distribuées. La combinaison de la sensibilisation de la population ainsi que la distribution gratuite des masses de la MILD ont un effet positif sur la survie des enfants de moins de 5 ans [16]. La réduction de la morbi-mortalité liée au paludisme chez les enfants de moins de 5 ans peut être obtenue en combinant l'usage de la MILD à l'insecticide aérosol d'usage domestique. Cette association est plus efficace que chacune de ces interventions utilisée séparément [17]. La possession et l'utilisation de la MILD permet aux mamans de contribuer à la prévention du paludisme chez les enfants de moins de 5 ans. L'utilisation de la MILD est influencée par le niveau d’éducation de la mère. Le développement des stratégies de distribution et de l'utilisation des MILD est utile pour accroître le contrôle du paludisme [18]. Il existe un écart entre l'acquisition de la MILD et son utilisation ainsi que l'adhérence à leur utilisation dans les familles ayant des enfants de moins de 5 ans. Les aspects sociaux des familles doivent être explorés pour se faire une idée sur l'adhérence à l'usage de la MILD [19]. Une étude menée dans une région endémique au paludisme à l'Est de la province de la Zambie dans 516 ménages des fermiers ruraux où la distribution gratuite des MILD supplémentaire leur a permis non seulement d’épargner de l'argent mais aussi de réduire de 40 à 42% le risque de contracter le paludisme [20]. La participation des parents dans les interventions de lutte contre le paludisme, l'analyse de la perception et des représentations qu'ils font de la moustiquaire contribuent à la réduction de la prévalence de la morbi-mortalité liée au paludisme chez les enfants de moins de 5 ans en Afrique sub-saharienne [21]. Brentlinger a trouvé que l'utilisation de la MILD est une stratégie utile pour la prévention contre le paludisme. La sélection de groupes à risque ainsi que la promotion de la moustiquaire constituent un mécanisme très important. Sur l'ensemble de sa population d’étude, 48% de ménages possédaient au moins une moustiquaire et 19,6% de cette proportion avaient une moustiquaire qui était traitée à l'insecticide six mois avant son étude [22]. Au Mali, Dicko a trouvé que la combinaison du TPI et l'utilisation de la MILD contribue à la prévention contre le paludisme dans les régions à faibles couverture en MILD chez les femmes enceintes et chez les enfants de moins de 5 ans [23]. L'impact de la MILD dans la prévention du paludisme peut être minimisé si elle n'est pas utilisée pour les populations vulnérables. Beaucoup de femmes enceintes et des enfants de moins de 5 ans n'utilisent pas la MILD. Parmi les facteurs qui constituent une barrière aux MILD constituent un important outil pour le contrôle du paludisme. Elles permettent de réduire la prévalence du paludisme parmi les enfants au cours de leur première année. Aussi, au cours de la deuxième année, le taux du paludisme clinique chute. Néanmoins, quelques facteurs dont la faible motivation de la communauté à dormir sous la MILD peuvent expliquer la hausse de la prévalence de cette maladie [24]. La couverture universelle en MILD demeure un défi majeur dans la prévention du paludisme en Afrique. L'utilisation précoce de la MILD pendant l'enfance constitue un facteur protecteur dans la mortalité infantile. Cependant, la prévention individuelle avec la MILD ne suffit pas pour réduire la prévalence de la malaria dans les régions de haute endémicité [25].

## Méthodes

Nous avons mené une étude descriptive transversale. Nos données ont été collectées auprès des ménages ayant au moins un enfant âgé de moins de 5 ans dans la zone de santé de Mumbunda. La population d’étude était constituée par l'ensemble de la population ayant au moins un enfant de moins de 5 ans de la zone de santé de Mumbunda. Le choix pour cette zone de santé peut se justifier par le fait qu'elle constitue une zone où s'est déroulée la campagne de distribution gratuite de MILD de l'avril 2012. Cette étude pourra contribuer à documenter le niveau d'utilisation de la moustiquaire chez les enfants de moins de 5 ans. La zone de santé de Mumbunda est l'une des onze zones de santé (ZS) du district sanitaire de Lubumbashi. C'est une ZS urbano-rurale et sa population totale est de 247.174 habitants sur une superficie de 30km^2^, soit une densité de 8.239habitants au km^2^. La morbidité dûe au paludisme chez les enfants de moins de 5ans représente 43,2% de l'ensemble des pathologie [26]. Nous avions retenu le ménage comme unité d'observation. Nous avons considéré 59.3% comme proportion de ménages ayant utilisé la MILD au Katanga [13]. La formule [27] suivante était utilisée pour calculer la taille de notre échantillon: n= taille d’échantillon requise z_α_= niveau de confiance à 95% (pour α=5%, la valeur de z0,05=1,96) p=pourcentage d'utilisation dans la population i= la précision désirée à 5% Après calcul, notre échantillon était de 369 ménages et nous avons ajouté une marge d'erreur 10% à cause d’éventuel refus et notre taille de l’échantillon a été ramenée à 410 ménages. Le dénombrement des ménages nous a donné pour l'ensemble de la zone 36970 ménages ayant au moins un enfant de moins de 5 ans. Suite à notre pas de sondage (36970/410), nous avons tiré un chiffre au hasard à l'aide de la table des nombres aléatoires entre 1 et 90 et nous avons procédé au tirage systématique de tous les ménages [28]. Les logiciels Epi-info 3.5.3 et Excel 2007 ont été utilisés pour la saisie, l'encodage et l'analyse des données. Les variables utilisées dans notre étude sont les suivantes: l'utilisation de la moustiquaire imprégnée d'insecticide à longue durée, raison d'utilisation de la MILD chez les enfants de moins de 5 ans, pratique du répondant à la première utilisation, différentes sources utilisées ayant expliqué l'usage de la moustiquaire, raisons de la non possession de la moustiquaire.

## Résultats

Parmi les ménages qui avaient déclaré possédaient la MILD 13,1% (n=54) des répondant avaient déclaré les utilisé chez les enfants de moins de 5ans et 80,2% pour tout le monde (Tableau 1). Les répondants savaient déclarer à 79,8% avoir utilisé la MILD pour se protéger contre la malaria et à 66,3% pour se protéger contre les piqures des moustiques (Tableau 2). La MILD a été étalée à l'ombre pendant 24h avant d’être utilisée pour la première fois par 77,9% des ménages (Tableau 3). Les répondants avaient déclaré à 15,3%(n=63) avoir reçu les conseils des médias pour l'usage de la moustiquaire. Et le personnel médical était la source la plus importante pour expliquer le mode d'emploi de la MILD pour 51,2% des ménages (Tableau 4). LeTableau 5 signale que la raison la plus importante de non possession de moustiquaire était le manque d'argent (17 ménages).

**Tableau 1:** Personnes qui avaient utilisé les MILD

MILD utilisée	Effectif	Pourcentage
Enfant de 0 à5ans	54	13,1
Enfant de plus de 5ans/adulte	20	4,9
Femme enceinte	4	1,0
Tout le monde	329	80,2
Autres	3	0,7

**Tableau 2:** Raisons d'utilisation de la MILD chez les enfants de moins de 5 ans

Raisons d'utilisation de la MILD	Fréquence	Pourcentage
Protection contre la malaria	327	79,8
Par habitude	9	2,2
Protection contre les piqures des moustiques	272	66,3
Protection contre le froid	4	1,0
Autres à préciser	5	1,2

**Tableau 3:** Pratique du répondant avant la 1^ère^ utilisation de la MILD

Pratique	Fréquence	Pourcentage
Etaler au soleil	51	14,3
Etaler à l'ombre pendant 24h	279	77,9
Laver à l'eau	14	3,9
Rien	14	3,9
Total	358	100%

**Tableau 4:** Différentes sources d'information ayant expliqué l'usage de la MILD

Source d'information	Fréquence	Pourcentage
Média	63	15,4
Relais communautaire	69	16,8
Personnel médical	210	51,2
Voisin	30	7,3
Vendeur	15	3,7
Famille	10	2,4
Notice	13	3,2

**Tableau 5:** Raisons de la non possession de la MILD par les ménages

Raisons	Effectifs	Pourcentage
Ne connait pas de MILD	14	18,2
Manque d'argent	17	22,0
Je ne sais pas où on vend	5	6,5
Stock épuisé	14	18,2
Chaleur dans la moustiquaire	8	10,4
Pas de moustique à la maison	7	9,0
Absence et retard lors de la distribution	1	1,2
Mauvaise qualité	4	5,2
Inadaptée au lit	4	5,2
Autres	3	3,9

## Discussion

La majorité des ménages utilisaient la MILD pour tout le monde (80,2%) et ceci peut être expliqué par le fait que la population a jugé bon de protéger tout le monde par ce moyen y compris les plus vulnérables qui sont les enfants. Pour ce qui est des raisons avancées par les répondants, la protection des enfants de moins de cinq ans contre le paludisme a été la raison principale (79,8%). C'est le cas avec Akilimali qui a trouvé 81,6% [29]. Dans notre étude (77,9%) comme dans l'enquête réalisée par l'Unicef Katanga (55,1%) [13]. L’étalage de la MILD à l'ombre a constitué la précaution dans la majorité des ménages avant l'utilisation de celle-ci. Ceci pourrait s'expliquer par la sensibilisation de la population sur cette pratique à travers le personnel de santé et les médias. La population était beaucoup plus informée à partir des personnels soignants (51,2%) et ceci pourrait être expliqué par le fait que les séances d’éducation sanitaire étaient plus animées par le thème d'utilisation de la moustiquaire imprégné. Chez les ménages qui ne possédaient pas de MILD, les raisons les plus avancées étaient le manque d'argent (22,0%). Il en est de même avec l’étude menée par Ndo au Cameroun où la même raison a été évoquée par les répondants (24,4%) [30]. Osero dans son étude a noté qu'en plus du manque d'argent il y a l'ignorance quant aux précautions d'usage par les chefs de ménages comme motifs de non possession [31].

## Conclusion

Ces résultats interpellent en ce qui concerne la sensibilisation de la communauté sur l'utilisation correcte de la moustiquaire imprégnée comme moyen de se protéger contre le paludisme surtout dans le pays en voie de développement. Ainsi pour améliorer l'utilisation de la MILD chez les enfants de moins de 5 ans, nous recommandons à tous les responsables de la santé de renforcer la distribution gratuite de la moustiquaire imprégnée dans la paquet minimum des activités du centre de santé qui est la porte d'entrée des malades dans notre système de santé.

### Etat des connaissance sur le sujet

- L'utilisation des moustiquaires est utile pour accroitre le contrôle du paludisme.
- Le manque des moyens financiers est souvent la raison de manque des moustiquaires dans les ménages.
- La protection des enfants de moins de cinq ans contre le paludisme est souvent la raison expliquant l'utilisation des moustiquaires.

### Contribution de notre étude a la connaissance

- Par rapport aux moustiquaires, la population est beaucoup plus informée auprès des personnels soignants.
- Pour qu'il y ait amélioration de l'utilisation de la moustiquaire imprégnée, il faudrait que les responsables de la santé renforcent la distribution gratuite au niveau des centres de santé.
- La nécessité de renforcer la sensibilisation sur l'utilisation correcte de la moustiquaire imprégnée.
